# Thermometrical Report of the Climate at Aiken, S. C.

**Published:** 1851-06

**Authors:** Thos. D. Mütter


### *Thermometrical Report of the Climate at Aiken, S. C.*

*May* 17, 1851.

To the Editors of the Medical Examiner :

GENTLEMEN,—I enclose a very interesting and valuable "table," prepared by my intelligent friend and patient, Mr. B., of this city, who passed several weeks at *Aiken, South Carolina.*

As this village is becoming a favorite winter resort, for invalids affected with pulmonary disease, any information bearing upon its climate, must prove useful to the profession.

Very truly yours,

THOS. D. MÜTTER.

*The Thermometer, at Aiken, S. C. winter of* 1850 *and* '51. (*Northern Exposure reached by no sun during the whole season.*)

| 1850. | 7 A.M. | | 12 M. | | 5 P.M. | |
|---|---|---|---|---|---|---|
| Dec. 23 | 42 | clear | | | | |
| " 24 | 26 | " | 44 | clear | 37 | clear |
| " 25 | 33 | cloudy | 49 | cloudy | 45 | cloudy |
| " 26 | 42 | " | 56 | clear | 49 | clear |
| " 27 | 38 | clear | 56 | " | 48 | rain |
| " 28 | 42 | rain | 48 | rain | 52 | cloudy |
| " 29 | 42 | clear | 48 | cloudy | | " |
| " 30 | 42 | cloudy | 48 | " | | " |
| " 31 | 36 | rain | 38 | " | 38 | " |
| 1851. | | | | | | |
| Jan. 1 | 40 | cloudy | 52 | clear | | " |
| " 2 | 39 | rain | 39 | rain | | rain & sleet |
| " 3 | 31 | clear 3 in. snow | 42 | clear | 38 | clear |
| " 4 | 37 | clear | 49 | " | | " |
| " 5 | 32 | clear | 54 | " | 46 | cloudy |
| " 6 | 37 | " | 55 | " | | clear |
| " 7 | 48 | " | 60 | " | | " |
| " 8 | 38 | " | 58 | " | 59 | " |
| " 9 | 48 | cloudy | 58 | rain | 55 | rain |
| " 10 | 49 | clear | 59 | cloudy | | cloudy |
| " 11 | 47 | cloudy | 56 | clear | 49 | clear |
| " 12 | 44 | clear | 55 | " | 50 | " |
| " 13 | 36 | " | 54 | " | 48 | " |
| " 14 | 28 | " | 52 | " | 48 | " |
| " 15 | 32 | " | 59 | " | 48 | " |
| " 16 | 54 | cloudy | 62 | cloudy | 50 | " |
| " 17 | 55 | clear | 68 | clear | 50 | " |
| " 18 | 44 | cloudy | 39 | rain | | rain |
| " 19 | 32 | " | 33 | cloudy | 33 | " |
| " 20 | 31 | rain | 47 | " | | cloudy |
| " 21 | 30 | clear | 50 | clear | 44 | clear |
| " 22 | 44 | " | 60 | | | |
| " 23 | 50 | rain | 46 | rain | 44 | " |
| " 24 | 44 | cloudy | 52 | clear | 49 | rain |
| " 25 | 37 | clear | 58 | " | 52 | clear |
| " 26 | 50 | cloudy | 59 | cloudy | 57 | " |
| " 27 | 52 | clear | 59 | clear | 50 | " |
| " 28 | 29 | " | 60 | " | 50 | " |
| " 29 | 30 | " | 55 | " | 50 | " |
| " 30 | 33 | " | 58 | " | 48 | " |
| " 31 | 25 | " | 37 | " | 32 | " |
| Feb. 1 | 30 | " | 40 | " | 35 | " |
| " 2 | 44 | " | 62 | " | 55 | " |
| " 3 | 55 | rain | 62 | rain | 56 | " |
| " 4 | 54 | cloudy | 60 | cloudy | 55 | " |
| " 5 | 58 | clear | 62 | clear | 55 | " |
| " 6 | 60 | " | 65 | " | 60 | " |
| " 7 | 55 | " | 62 | " | 60 | " |
| " 8 | 60 | cloudy | 70 | cloudy | 65 | " |
| " 9 | 64 | " | 68 | " | 65 | " |
| " 10 | 67 | rain | 65 | " | 65 | " |
| " 11 | 50 | clear | 60 | clear | 58 | " |
| " 12 | 34 | " | 54 | " | 54 | " |
| " 13 | 40 | cloudy | 52 | cloudy | 50 | cloudy |
| " 14 | 40 | rain | 50 | rain | 48 | rain |
| " 15 | 62 | cloudy | 65 | cloudy | 60 | cloudy |

| | 7 A.M. | | 12 M. | | 5 P.M. | |
|---|---|---|---|---|---|---|
| Feb. 16 | 40 | clear | 55 | clear | 50 | clear |
| " 17 | 30 | " | 55 | " | 40 | " |
| " 18 | 41 | cloudy | 52 | cloudy | | |
| " 19 | 39 | clear | 60 | clear | 59 | clear |
| " 20 | 49 | cloudy | 60 | cloudy | 58 | cloudy |
| " 21 | 59 | rain | 63 | rain | 59 | rain |
| " 22 | 50 | cloudy | 67 | clear | | |
| " 23 | 53 | " | 68 | " | 64 | clear |
| " 24 | 61 | clear | 79 | " | | " |
| " 25 | 42 | " | 64 | " | 56 | " |
| " 26 | 37 | " | 64 | " | 58 | " |
| " 27 | 47 | " | 68 | " | 65 | " |
| " 28 | 56 | " | 72 | " | | " |
| Mar. 1 | 44 | " | 58 | " | | " |
| " 2 | 44 | " | 56 | cloudy | | " |
| " 3 | 37 | " | 50 | clear | 46 | clear |
| " 4 | 26 | " | 54 | " | | " |
| " 5 | 36 | " | 68 | " | | " |
| " 6 | 58 | rain | 69 | cloudy | 70 | clear |
| " 7 | 60 | " | 70 | rain | 48 | rain |
| " 8 | 42 | cloudy | 50 | clear | 48 | clear |
| " 9 | 32 | clear | 49 | " | | " |
| " 10 | 34 | " | 52 | " | 47 | " |
| " 11 | 36 | " | 58 | " | 58 | " |
| " 12 | 39 | " | 65 | " | 58 | " |
| " 13 | 39 | " | 66 | " | | " |
| " 14 | 49 | cloudy | 70 | " | | " |
| " 15 | 50 | clear | 70 | " | 70 | " |
| " 16 | 58 | " | 70 | " | 68 | " |
| " 17 | 50 | " | 65 | " | 50 | " |
| " 18 | 44 | " | 58 | " | 52 | " |
| " 19 | 39 | " | 57 | " | 50 | " |
| " 20 | 33 | " | 54 | " | 57 | " |
| " 21 | 33 | " | 62 | " | 62 | " |
| " 22 | 50 | cloudy | 53 | rain | | " |
| " 23 | 48 | " | 62 | clear | 55 | " |
| " 24 | 43 | " | 61 | " | 57 | " |
| " 25 | 39 | clear | 66 | " | 59 | " |
| " 26 | 40 | " | 70 | " | 69 | " |
| " 27 | 53 | " | 70 | " | 68 | " |
| " 28 | 52 | " | 76 | " | 70 | " |
| " 29 | 57 | cloudy | 68 | rain | | rain |
| " 30 | 58 | " | 70 | cloudy | 62 | " |
| " 31 | 58 | rain | 68 | rain | 62 | " |
| Apr. 1 | 58 | cloudy | 71 | clear | 67 | cloudy |
| " 2 | 60 | rain | 70 | rain | 62 | " |
| " 3 | 54 | clear | 63 | clear | 58 | " |
| " 4 | 46 | " | 65 | " | 64 | clear |
| " 5 | 58 | rain | 73 | cloudy | 70 | cloudy |
| " 6 | 59 | clear | 69 | clear | 70 | clear |
| " 7 | 50 | " | 74 | cloudy | 70 | rain |
| " 8 | 64 | rain | 66 | " | 64 | clear |
| " 9 | 43 | clear | 58 | clear | 58 | " |
| " 10 | 44 | " | 64 | " | | " |
| " 11 | 51 | " | 69 | " | 68 | " |
| " 12 | 49 | " | 72 | " | 65 | " |
| " 13 | 56 | " | | | | |

